# The role of versican G3 domain in regulating breast cancer cell motility including effects on osteoblast cell growth and differentiation *in vitro* – evaluation towards understanding breast cancer cell bone metastasis

**DOI:** 10.1186/1471-2407-12-341

**Published:** 2012-08-03

**Authors:** William Weidong Du, Ling Fang, Weining Yang, Wang Sheng, Yaou Zhang, Arun Seth, Burton B Yang, Albert J Yee

**Affiliations:** 1 Sunnybrook Research Institute, Toronto, ON, Canada; 2 Department of Laboratory Medicine and Pathobiology, University of Toronto, Toronto, ON, Canada; 3 Centre for the Study of Bone Metastasis, Odette Cancer Centre, and Holland Musculoskeletal Program, Sunnybrook Health Sciences Centre, Division of Orthopaedic Surgery, Department of Surgery, University of Toronto, Ontario, Canada; 4 Sunnybrook Health Sciences Centre and Centre for the Study of Bone Metastasis, Odette Cancer Centre, Department of Surgery, University of Toronto, 2075 Bayview Avenue, Rm. MG 371-B, Toronto, ON, M4N 3 M5, Canada

**Keywords:** Metastasis, Cell differentiation, Apoptosis, Invasion, Versican, G3 domain

## Abstract

**Background:**

Versican is detected in the interstitial tissues at the invasive margins of breast carcinoma, is predictive of relapse, and negatively impacts overall survival rates. The versican G3 domain is important in breast cancer cell growth, migration and bone metastasis. However, mechanistic studies evaluating versican G3 enhanced breast cancer bone metastasis are limited.

**Methods:**

A versican G3 construct was exogenously expressed in the 66c14 and the MC3T3-E1 cell line. Cells were observed through light microscopy and viability analyzed by Coulter Counter or determined with colorimetric proliferation assays. The Annexin V-FITC apoptosis detection kit was used to detect apoptotic activity. Modified Chemotactic Boyden chamber migration invasion assays were applied to observe tumor migration and invasion to bone stromal cells and MC3T3-E1 cells. Alkaline phosphatase (ALP) staining and ALP ELISA assays were performed to observe ALP activity in MC3T3-E1 cells.

**Results:**

In the four mouse breast cancer cell lines 67NR, 66c14, 4T07, and 4T1, 4T1 cells expressed higher levels of versican, and showed higher migration and invasion ability to MC3T3-E1 cells and primary bone stromal cells. 4T1 conditioned medium (CM) inhibited MC3T3-E1 cell growth, and even lead to apoptosis. Only 4T1 CM prevented MC3T3-E1 cell differentiation, noted by inhibition of alkaline phosphatase (ALP) activity. We exogenously expressed a versican G3 construct in a cell line that expresses low versican levels (66c14), and observed that the G3-expressing 66c14 cells showed enhanced cell migration and invasion to bone stromal and MC3T3-E1 cells. This observation was prevented by selective EGFR inhibitor AG1478, selective MEK inhibitor PD 98059, and selective AKT inhibitor Triciribine, but not by selective JNK inhibitor SP 600125. Versican G3 enhanced breast cancer cell invasion to bone stromal cells or osteoblast cells appears to occur through enhancing EGFR/ERK or AKT signaling. G3 expressing MC3T3-E1 cells showed inhibited cell growth and cell differentiation when cultured with TGF-β1 (1 ng/ml), and expressed enhanced cell apoptosis when cultured with TNF-α (2 ng/ml). Enhanced EGFR/JNK signaling appears to be responsible for G3 enhanced osteoblast apoptosis and inhibited osteoblast differentiation. Whereas repressed expression of GSK-3β (S9P) contributes to G3 inhibited osteoblast growth. Versican G3 functionality was dependent on its EGF-like motifs. Without the structure of EGF-like repeats, the G3 domain would not confer enhancement of tumor cell migration and invasion to bone with concordant inhibition of osteoblast differentiation and promotion of osteoblast apoptosis.

**Conclusions:**

Versican enhances breast cancer bone metastasis not only through enhancing tumor cell mobility, invasion, and survival in bone tissues, but also by inhibiting pre-osteoblast cell growth, differentiation, which supply favorable microenvironments for tumor metastasis.

## Background

The predilection for breast cancer to metastasize to bone has been recognized for more than 50 years. However, the underlying mechanisms which regulate the haptotactic migration of breast cancer cells to bone have not been firmly established. Metastasis to bone occurs frequently in most advanced breast cancers, accompanied by complications in the form of skeletal related events (such as bone fracture), dramatically reducing the patient’s quality of life [1]. As with many other metastatic cancers, breast cancer cells must take a series of steps to metastasize to bone. These include detaching from the primary tumor, invading the surrounding tumor stroma, intra-vasating into local blood vessels, surviving in the bloodstream, and colonizing the bony tissues, thereby forming metastatic tumors [2,3]. The intrinsic metastatic propensity of breast cancer cells, such as loss of cell polarity, reduction of cell-cell and cell-matrix adhesion, which support detachment, migration and invasion of tumor cells, is a major determinant of metastatic efficiency [4]. The importance of the bone microenvironment in determining tumor cell colonization and growth is also broadly accepted, commonly named the “seed and soil” theory [4-6]. Specific aspects of both breast cancer cells and the bone microenvironment are likely important contributors to the development of bone metastasis [5,6].

Tumor cell-autonomous changes alone are not sufficient to allow tumor progression and metastasis to occur [7]. It is well known that the supportive “stroma” around the solid tumor, consisting of specific extracellular matrix (ECM) components, plays an important role in activating the tumor microenvironment at the primary and second tumor sites [5,8]. The interaction between tumor cells and the ECM, which is mediated by cell-cell contact, growth factor signaling and paracrine cytokine activity facilitates tumor cell outgrowth, invasion and metastasis [4,9].

Versican is a member of the large aggregating chondroitin sulfate proteoglycans and belongs to the lectican family. To date, four isoforms of versican (V0, V1, V2 and V3) have been identified in various tissues. Structurally all versican isoforms include an N-terminal G1 domain, a glycosamingoglycan (GAG) attachment region, and a C-terminus containing a selectin-like (G3) domain. With exception is the V3 isoform, which has no GAG region [10]. The G3 domain contains two epidermal growth factor (EGF)-like repeats, a lectin-like motif (carbohydrate recognition domain), and a complement binding protein (CBP)-motif. Given their ubiquitousness and high degree of conservation, it is likely that the G1 and G3 domains play a vital role in proteoglycan function [11]. There is an increasing recognition of the importance of the G3 domain to tumor growth, motility, and metastasis [12-15].

Versican is detected in the interstitial tissues at the invasive margins of breast carcinoma and in the elastic tissues associated with tumor invasion [12]. Immunolocalization of versican in breast tumors, including infiltrating ductal carcinoma, has been reported [16]. The high expression of versican in human breast tumor appears prognostic, is predictive of relapse, and negatively impacts overall survival rates [17,18]. Direct evidence of versican functions have been obtained by ectopic expression of full-length versican [19-21]. Previous studies shows that the activity of the versican G3 domain is important in breast cancer cell growth, migration and metastasis [12]. Versican G3 domain enhanced breast cancer progression, metastasis, chemical reagent (i.e. chemotherapy) resistance, and tumor cell self-renewal is modulated by the up-regulation of Epidermal Growth Factor Receptor (EGFR)-mediated signaling [22-24].

In our previous work we characterized the expression of versican in murine mammary epithelial tumor cell lines 67NR, 66c14, 4T07, and 4T1 (which were derived from a single spontaneous arising mammary tumor from Balb/cfC3H mice) [25,26]. Versican was highly expressed in the 4T1 cell line which is one of the very few cell lines of any origin that spontaneously metastasize to bone. This closely mimicks Stage IV human breast cancer which hematogeneously metastasizes to the lung, liver, bone, and brain [27]. Most interestingly, exogenous expression of the versican G3 fragment in a mammary carcinoma 66 cl4 cell line (a cell line that expresses low levels of versican and normally metastasizes to the lung but not the bone) was sufficient not only to promote local tumor growth but also to enhance metastasis to bone from the mammary fat pad [22]. In order to investigate the potential mechanisms through which versican expression promoted breast cancer cell bone metastasis, we exogeneously expressed a versican G3 domain in mouse breast cancer cell line 66c14 and mouse pre-osteoblast-like cell line MC3T3-E1. The purpose of this study was to determine the effects of the versican G3 domain on breast cancer cell invasion and migration to primary bone stromal and pre-osteoblast MC3T3-E1 cells. The effects of G3 on bone stromal and pre-osteoblast cell growth, differentiation, and apoptosis would also be evaluated.

## Methods

### Material supplies

The polyclonal antibody against pEGFR was obtained from Santa Cruz Biotechnology. The polyclonal antibodies against pSAPK/JNK and pAKT were obtained from Cell Signaling. The polyclonal antibodies against versican V1 isoform, Glycogen synthase kinase-3 β serine-9 phosphorylation (GSK-3β, S9P), were obtained from Abcam. EGF, selective EGFR inhibitor AG 1478, selective MEK inhibitor PD 98059, selective pSAPK/JNK inhibitor SP 600125, the monoclonal antibody against β-actin, and the Alkaline phosphatase kits used in the study were obtained from Sigma. Selective AKT inhibitor Triciribine was from Calbiochem. Horseradish peroxidase-conjugated goat anti-mouse IgG and horseradish peroxidase-conjugated goat anti-rabbit IgG were obtained from Bio-Rad. Immunoblotting was performed using the ECL Western blot detection kit. Cell Proliferation Reagent WST-1 was obtained from Roche Applied Science.

### Cell culture

The pre-osteoblast-like cell line - MC3T3-E1 was cultured in alpha modified Eagle’s medium (AMEM) supplemented with 10% fetal calf serum, penicillin (100 U/ml) and streptomycin (100 μg/ml) and maintained at 37°C in a humidified atmosphere of 5% CO_2_. Mouse mammary tumor cell lines 67NR, 66c14, 4T07, 4T1 were cultured in DMEM media, which were supplemented with 10% fetal calf serum, penicillin (100 U/ml) and streptomycin (100 μg/ml) and maintained at 37°C in a humidified atmosphere of 5% CO_2_. In selected experiments, cell suspensions were cultured with EGF (20 ng/ml), EGFR inhibitor AG 1478 (2 μM), selective MEK inhibitor PD 98059 (50 μM), selective SAPK/JNK inhibitor SP 600125 (100 nM), and selective AKT inhibitor Triciribine (2 μM).

### Exogenous expression of versican G3 construct in MC3T3-E1 and 66 C14 cell lines

The pcDNA1 - G3 construct and pcDNA1 - G3 fragment lacking the EGF-like motifs (G3ΔEGF) construct were generated by our group [28]. The mouse pre-osteoblast-like cell line MC3T3-E1 and mouse mammary tumor cell line 66c14, were transfected with pcDNA1-vector, G3 construct, and G3ΔEGF construct, or the control vector. Three days after transfection, Geneticin was added to the growth medium at a concentration of 1 mg/ml, and the cells were maintained in this medium until individual colonies were large enough for cloning. Chemically selected stable cell lines were maintained in culture medium containing 0.5 mg/ml Geneticin or stored in liquid nitrogen.

### Cell proliferation assays

Versican G3- and ×vector-transfected MC3T3-E1 cells (2 × 10^4^ cells) were seeded onto 6-well dishes in 10% FBS/AMEM medium and maintained at 37°C overnight. Cells were harvested daily and cell number was counted under light microscope. Cell proliferation assays were also performed with a colorimetric proliferation assay (Cell Proliferation Reagent WST-1). Versican G3 and control vector transfected MC3T3-E1 (2 × 10^3^ cells/well) cells were cultured in 100 μl FBS/AMEM medium in 96 wells tissue culture microplates. The absorbance of the samples against a background blank control was measured daily for 5 days by a microplate (ELISA) reader. In selected experiments, cell suspensions were cultured with TGF-β (1 ng/ml), selective SAPK/JNK inhibitor SP 600125 (100 nM).

### Cell viability assays

G3 and vector-transfected MC3T3-E1 (2 × 10^5^) were cultured in 10% FBS/DMEM medium in culture dishes and maintained at 37°C for 12 hours. After cell attachment, we changed the medium to serum free DMEM medium or 10% FBS/DMEM medium containing 2 ng/ml TNF-α. Cells were harvested daily and cell number was analyzed by Coulter Counter. Cell survival assays were also performed with colorimetric proliferation assays (Cell Proliferation Reagent WST-1). Versican G3 and control vector transfected MC3T3-E1 (1 × 10^4^ cells/well) were inoculated and cultured in 10% FBS/DMEM medium in 96 well culture dishes for 12 hours. After cell attachment, we changed the medium into serum free DMEM medium or 10% FBS/DMEM medium containing 2 ng/ml TNF-α for 4 days and then cultured cells with 10 μl WST-1 reagents for 4 hours. The absorbance of the samples against a background blank control was measured by a microplate reader.

### Annexin V assays

An Annexin V-FITC apoptosis detection kit (Biovision Inc, Mountain View, CA, USA) was used to detect apoptotic activity. Cells (1 × 10^6^) were collected and resuspended in binding buffer. Annexin V-FITC and propidium iodide were added to each sample and incubated in the dark for 5 minutes. Annexin V-FITC binding was determined by flow cytometry (Ex = 488 nm; Em = 530 nm) using FITC signal detector (FL1) and propidium staining by the phycoerythrin emission signal detector (FL2).

### Cell migration assays

Modified chemotactic Boyden chamber migration assays: This assay was performed using 24-well cell culture plates and a 3 μm cell culture insert. The tibias and femora were harvested from Balb/c mice, crushed and digested with a solution of DMEM containing collagenase type II (6 mg/ml) and dispase II (8 mg/ml) for 60 minutes. The cell suspension was filtered through a 70 μm nylon filter and washed three times by centrifugation in DMEM. The cell pellet was resuspended in DMEM, 10% FBS and maintained at 37°C overnight. After 12–16 h of culture, these cells were allowed to form a confluent monolayer in the bottom well of Transwell migration chambers (5 × 10^4^). The medium was removed and washed with PBS, followed by culturing in 600 μl 10% DMEM with or without 2.0 μM AG 1478, 50 μM PD 98059 at 37°C for an additional incubation time of 2 hours. 1 × 10^5^ cells were gently injected into each filter insert (upper chamber) and then incubated at 37°C for 4 h. The filter inserts were removed from the chambers, fixed with methanol for 5 minutes, and stained with Harris’ Haemotoxylin for 20 minutes. Migrating cells were stained blue. Migration experiments were performed in triplicate and were counted in three fields of views/membrane.

The cell migration assay was also performed with MC3T3-E1 cells (5 × 10^4^) loaded in the bottom well of the Transwell migration chambers.

### Cell invasion assays

Modified chemotactic Boyden chamber invasion assays: This assay was performed using 24-well cell culture plates and an 8 μm cell culture insert. After culturing the bone stromal cells or MC3T3-E1 cells (5 × 10^4^) in the bottom well of Transwell migration chambers for 12 h, the medium was removed and the cultures were washed with PBS, followed by 100 μl diluted matrigel (1 mg/ml) filling in the upper chamber and 600 μl of 10% FBS/DMEM medium in lower chamber with the Transwell subsequently incubated at 37°C for 4 h. Cells (1 × 10^5^) in 100 μl serum free DMEM medium with were gently injected into each filter insert (upper chamber) and then incubated at 37°C for 24 – 72 h. The filter inserts were removed from the chambers, fixed with methanol for 5 minutes, and stained with Harris’ Haemotoxylin for 20 minutes. Samples were subsequently washed, dried, and mounted onto slides for analysis using a light microscope. The invasive cells were stained blue and were counted in 6 fields of views/membrane.

### Alkaline phosphatase staining

The MC3T3-E1 cells were seeded at a density of 8 × 10^4^ cells/well on 6 well plates. Cells were maintained in 10% FBS/AMEM medium for 21 days. The medium was changed every 3 days. Before staining, the cells were fixed in 4% paraformaldehyde for 15 min at room temperature. After washing with PBS, the cells were incubated with a mixture of Naphthol AS-MX phosphate solution and diluted diazonium salt solution for 30 min. After washing, the plates were incubated in Mayer’s Hematoxylin solution for 10 min. The staining was evaluated under microscope.

### Alkaline phosphatase ELISA assay

Cells were treated with 0.2% Triton X-100 and harvested. Lysates were centrifuged and supernatants (10 μg protein) were incubated with 150 μl pNPP for 5 hours at room temperature in the dark. Absorbance at 405 nm was measured using a microplate reader, and ALP activity was calculated according to manufacturer’s instructions (Sigma Aldrich).

### Western blot analysis

Protein samples were subjected to sodium dodecyl sulfate-polyacrylamide gel electrophoresis (SDS-PAGE) on separating gel containing 7–10% acrylamide. Separated proteins were transblotted onto a nitrocellulose membrane in 1 × Tris/glycine buffer containing 20% methanol at 60 V for 2 hours in a cold room. The membrane was blocked in TBST (10 mM Tris-Cl, pH 8.0, 150 mM NaCl, 0.05% Tween 20) containing 5% non-fat dry milk powder (TBSTM) for 1 hour at room temperature, and then incubated with primary antibodies at 4°C overnight. The membranes were washed with TBST (3 × 30 minutes) and then incubated with appropriate horseradish peroxidase-conjugated secondary antibodies in TBSTM for 1 hour. After washing as above, the bound antibodies were visualized with an ECL detection kit.

## Results and discussion

### Effects of conditioned medium of mouse mammary tumor cells on MC3T3-E1 cell growth and differentiation

Breast cancer frequently metastasizes to bone, resulting in osteolytic lesions. These lesions, formed by increased osteoclastic activity and reduced osteoblastic activity, are reflected by decreases in both osteoid volume and osteoblastic surface [29,30]. It has been known that breast cancer cells communicate with osteoblasts and subsequently activate osteoclast activity. It has also been reported that breast cancer cells can induce apoptosis of osteoblast cells and bone marrow stromal cells [31,32]. Breast cancer cells also inhibit osteoblast cell differentiation *in vitro*. Conditioned medium (CM) of human breast cancer cell line MDA-MB-231 showed inhibitive effects on MC3T3-E1 mouse pre-osteoblast cell differentiation. TGF-β in the medium was identified as the main factor that caused the inhibition of MC3T3-E1 differentiation, motivating further evaluation in the present study [33].

**Figure 1:**
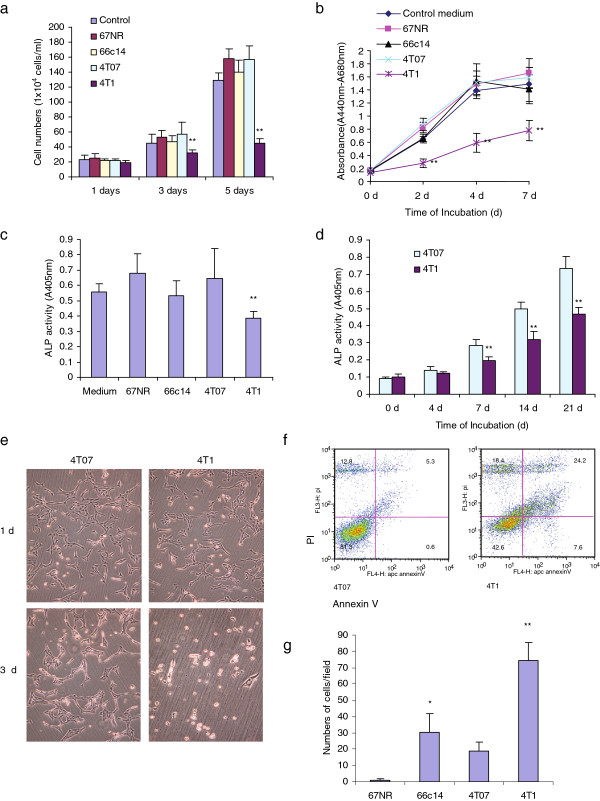
** The 4T1 CM inhibits MC3T3-E1 cell growth and differentiation, and enhances MC3T3-E1 cell apoptosis.(a)** MC3T3 cells (2 × 10^4^) were inoculated in 6-well culture dishes containing 10% FBS/AMEM and cultured for 12 h. After cell attachment, we changed the medium to 10% FBS/DMEM conditioned medium (CM) which had been pre-incubated with MC3T3-E1, 67NR, 66c14, 4T07, and 4T1 for 2 d, and kept culture for 5 days. Cells were harvested and counted under light microscopy every 2 days. *n* = 6, * *p* < 0.05, **p < 0.01, analyzed with *t*-test. **(b)** MC3T3-E1 cells (1 × 10^3^) were inoculated in 96-well culture dishes and cultured in 10%FBS/AMEM medium for 12 h. After cell attachment, we changed the medium to MC3T3-E1, 67NR, 66c14, 4T07, and 4T1 CM, and kept culture for 7 d. Proliferation assays performed with WST-1 Assays. All groups compared with control group, *n* = 8, * p < 0.05, ** p < 0.01, analyzed with *t*-test. **(c)** The MC3T3-E1 cells were seeded at 8 × 10^4^ cells/well in 6 well plates. Cells were maintained in MC3T3-E1, 67NR, 66c14, 4T07, and 4T1 CM for 21 days. The medium was changed every 3 d. After 21 d, cell lysates were processed to ALP ELISA Assay. All groups compared with control group, *n* = 4, * p<0.05, ** p<0.01, analyzed with *t*-test. **(d)** ALP ELISA Assay showed the ALP level of MC3T3-E1 cells cultured in 4T07 and 4T1 CM. Compared with 4T07 cells, *n* = 6, * p<0.05, ** p<0.01, analyzed with *t*-test. **(e)** MC3T3 cells (2 × 10^5^) were inoculated in 6-well culture dishes containing 10% FBS/AMEM and cultured for 12 h. After cell attachment, we changed the serum free DMEM medium which had been pre-incubated with 4T07 and 4T1 for 2 d, and then cultured the MC3T3 cells for 3 d. Cells were harvested and counted under light microscopy every 2 days. Typical pictures showed that the medium pre-incubated with 4T1 cells enhanced MC3T3-E1 cell apoptosis. **(f)** After cultured in to serum free DMEM medium which had been pre-incubated with 4T07, and 4T1 for 2 d, the MC3T3 cells were kept culture for 1 d. Cells were analyzed with Annexin V and propidium iodide staining using flow cytometry. **(g)** Modified chemotactic Boyden chamber cell invasion assays (48 h) indicated that 4T1 cell line showed highest invasive ability among the 4 mouse breast cancer cell lines. Compared with 4T07 cell line, *n* = 4, * p<0.05, ** p<0.01, analyzed with *t*-test.

In this study, we found that the growth of mouse pre-osteoblasts MC3T3-E1 cells were significantly inhibited by mouse mammary tumor cell line 4T1 conditioned medium (CM). Other mouse mammary tumor cell lines 67NR, 66c14 and 4T07 CM did not alter the proliferation of MC3T3-E1 cells (Figure 1a, Figure 1b and Additional file 1: Figure S1a). Only 4T1 CM prevented MC3T3-E1 cell differentiation, noted by inhibition of alkaline phosphatase (ALP) activity (Figure 1c). ALP ELISA Assay showed that the ALP levels of MC3T3-E1 cells cultured in 4T1 CM were significantly lower than that observed in 4T07 CM over 7, 14 and 21 days (Figure 1d, Additional file 1: Figure S1b). The 4T1 serum free CM could induce MC3T3-E1 cell apoptosis after 3 days of culture (Figure 1e and Figure 1f). Chemotactic chamber cell migration assays and cell invasion assays showed that 4T1 cells showed higher migration and invasion ability towards MC3T3-E1 cells and primary bone stromal cells (Additional file 1: Figure S1c, Figure 1g, Additional file 2: Figure S2a and Additional file 2: Figure S2b). To investigate the molecular determinants contributing to the invasive capacity of 4T1 cells to bone, we tested different molecules expressed in the four mouse mammary tumor cell lines. Through immunoblotting, we observed that the 4T1 cell expressed higher levels of the versican V1 isoform (250–300 kDa) (Additional file 1: Figure S1d).

Increased expression of the versican V0 and V1 isoforms have been reported in breast cancer and other malignant tumors, generally conferring poor prognosis [34,35]. The four mouse mammary tumor cell lines 67NR, 66c14, 4T07, and 4T1 were derived from a single spontaneous arising mammary tumor from Balb/cfC3H mice, whose tumorigenic and metastatic potential has been characterized [25-27]. Although they share a common origin, these cell lines are phenotypically heterogeneous in their metastatic behavior. The 4T1 cell line is one of the very few cell lines of any origin that spontaneously metastasizes to bone. This closely mimics Stage IV human breast cancer, which hematogeneously metastasizes to the lung, liver, bone, and brain. 66c14 cells appear to metastasize to the lung and liver via the lymphatic system [26]. 67NR cells fail to leave the primary site, while 4T07 cells are highly tumorigenic locally but fail to metastasize [25]. Cancer cell invasiveness towards bone stroma and the inhibitory effects observed in both pre-osteoblast cell growth and differentiation appear influenced by versican. Our *in vitro* study complements this understanding. Greater versican expression in 4T1 cells compared to other breast cancer cell lines may be associated with the predilection towards bone metastasis.

### Expression of versican G3 domain enhanced breast cancer cell migration and invasion

Versican interacts with its binding partners through its N- and C-terminal globular regions as well as its central GAG-binding region [36]. It is known to associate with a number of molecules in the extracellular matrix (ECM) including hyaluronan [37] fibronectin [38], P- and L-selectin, and various chemokines [36]. Versican also binds to the cell surface proteins epidermal growth factor receptor (EGFR) [36], P-selectin [14], CD44 [39] and integrin β1 [40]. Increasingly, experimental evidence and clinical data support the understanding that versican participates in cell adhesion, proliferation, migration, and angiogenesis. It plays a central role in normal tissue morphogenesis and maintenance, while contributing to the process of tumorigenesis [11,41]. Versican G3 enhances local breast cancer progression, systemic metastases, and influences chemotherapy effects on cancer cells. Cell stromal interactions involve VEGF and fibronectin [12]. We have also previously demonstrated the importance of EGF-like motifs to G3 functionality. However, the mechanisms by which G3 influence bone activity is poorly understood and results of the present study bridges that knowledge gap [22-24].

**Figure 2:**
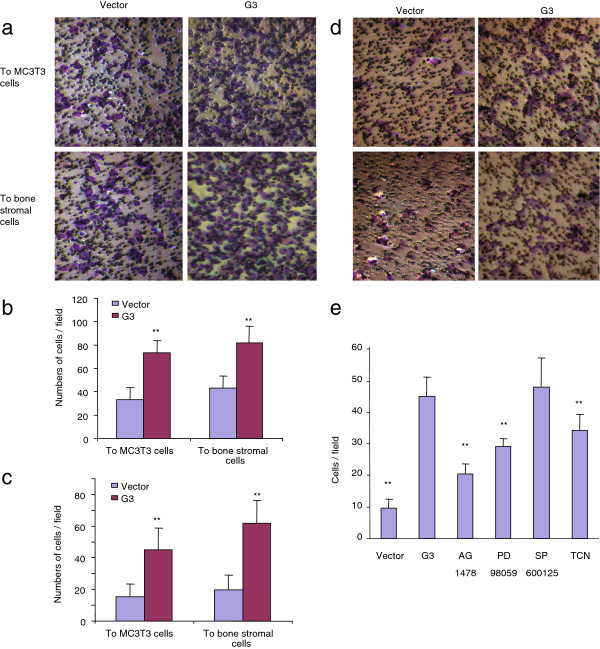
** Expression of versican G3 domain enhanced tumor cell migration and invasion to bone stromal cells and MC3T3-E1 cells.(a)** After culture the bone stromal cells or MC3T3-E1 cells in the bottom well of Transwell migration chambers for 12 h, vector- and G3- transfected 66c14 cells (1 × 10^5^) were loaded in the insert with 100 μl serum free DMEM medium and then incubated at 37°C for 4 hours. The migration cells were stained blue and were counted in 6 fields of views/membrane using a light microscope. Typical pictures showed that migration cells of vector- and G- transfected 66c14 cell migration to bone stromal cells and MC3T3-E1 cells after 4 hours. **(b)** Graph showed vector- and G3- transfected 66c14 cell migration to stromal cells and MC3T3-E1 cells. **, *p*<0.01. Error bars indicate SD (*n* = 6). (c) After culture the bone stromal cells or MC3T3-E1 cells in the bottom well of Transwell migration chambers for 12 h, vector- and G3- transfected 66c14 cells (1 × 10^5^) were loaded in the insert pre-set with Matrigel gel with 100 μl serum free DMEM medium and then incubated at 37°C for 48 hours. Graph showed vector- and G3- transfected 66c14 cell invading to stromal cells and MC3T3-E1 cells. **, *p*<0.01. Error bars indicate SD (*n* = 6). **(d)** Typical pictures showed that invasive cells of vector- and G- transfected 4T07 to bone stromal cells and MC3T3-E1 cells. **(e)** Graph showed that vector- and G3- transfected 66c14 cell invasion to MC3T3-E1 cells which were cultured in selective EGFR inhibitor AG1478 (2 μM), selective MEK inhibitor PD 98059 (50 μM), selective JNK inhibitor SP 600125 (100 nM), or selective AKT inhibitor Triciribine (2 μM). The invasive cells were stained blue and were counted in 6 fields of views/membrane using a light microscope. **, p<0.01. Error bars indicate SD (*n* = 6).

It seems that the over-expression of versican might be an important factor in conferring 4T1 cells with an enhanced ability to metastasize to bone. To further investigate the effects of versican on breast cancer bone metastasis, we exogenously expressed a versican G3 construct in one of the mouse mammary tumor cell line 66c14. After transfection, we found that the G3-expressing 66c14 cells showed enhanced cell migration and invasion to MC3T3-E1 cells (Figure 2a, Figure 2b, Figure 2c, and Figure 2d). We observed that versican G3 enhanced cell invasion could be prevented by selective EGFR inhibitor AG1478 (2 μM), selective MEK inhibitor PD 98059 (50 μM), and selective AKT inhibitor Triciribine (2 μM) (Figure 2e). However, these observed effects were not blocked by selective JNK inhibitor SP 600125 (100 nM). Enhanced EGFR/ERK or AKT signaling appears to be involved in G3’s ability to invade bone stromal and pre-osteoblast cells [22].

### Expression of versican G3 domain regulated MC3T3-E1 cell differentiation, growth and apoptosis

Although tumors are typically defined by their uncontrolled and invasive growth, some are supported by the surrounding stroma when metastasizing to distant organs. Tumor phenotype considers both local and systemic immune factors [42,43]. Specific cytokines and growth factors, such as transforming growth factor-β (TGF-β), tumor necrosis factor-α (TNF-α), have been implicated in influencing tumor-stromal connectivity both locally and from a systemic perspective [42,43].

In breast cancer, TGF-β signaling has been shown to reduce growth of the primary tumor but also to promote metastasis, indicating that the apparent effect of TGF-β depends on its cellular context [44]. It was reported to have a dual role in breast cancer progression. During the early stages of tumorigenesis, TGF-β inhibits tumor growth, but in advanced cancer it loses its growth inhibitive function, and continues to stimulate tumor cell metastasis [45]. Elevated plasma TGF-β was reported in advanced breast cancer, hepatocellular carcinoma, lung and prostate cancer patients and correlated with poor outcome [44]. Systemic TGFβ1 levels have been used as a surrogate of tumor load and/or response to therapy [46]. TGF-β is also abundant in bone matrix. It is released from bone matrix and is activated by osteoclastic re-absorption. TGF-β stimulates breast cancer cell to secrete other growth factors including Parathyroid Hormone related protein, contributing to breast cancer bone metastasis [47].

**Figure 3:**
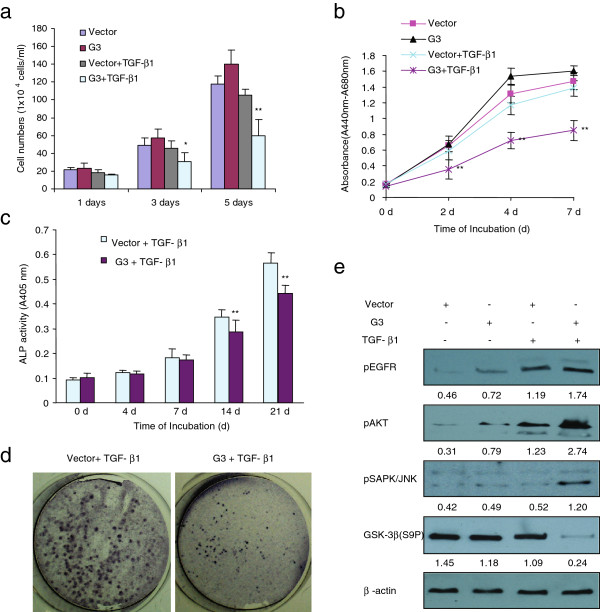
** Expression of versican G3 domain regulated MC3T3-E1 cells growth and differentiation.(a)** Vector- and G3- transfected MC3T3 cells (2 × 10^4^) were inoculated in 6-well culture dishes containing 10% FBS/AMEM and cultured for 12 h. After cell attachment, the cells were cultured with or without 1 ng/ml TGF-β1 for 5 d. Cells were also counted by light microscope in 1, 3, 5 days. *n* = 6, * p<0.05, ** p<0.01, analyzed with *t*-test. **(b)** The G3- and vector-transfected MC3T3-E1 cells (1 × 10^3^) were inoculated in 96-well culture dishes and cultured in AMDM medium containing 1 ng/ml TGF-β1 or not for 7 days. Proliferation assays performed with WST-1 assay. All groups compared with vector control cells, *n* = 8, * p<0.05, ** p<0.01, analyzed with *t*-test. **(c)** The vector- and G3-transfected MC3T3-E1 cells were seeded at 8 x10^4^ cells/well in a 6 well plate. Cells were maintained in 10% FBS/AMEM medium containing 1 ng/ml TGF-β1 for 21 days. The medium was changed every 3 days. Cells were lysised and processed to ALP ELISA Assay. All groups compared with vector control cells, *n* = 6, * p<0.05, ** p<0.01, analyzed with *t*-test. **(d)** Typical pictures showed ALP staining of vector- and G3-transfected MC3T3-E1 cells maintained in 10% FBS/AMEM medium containing 1 ng/ml TGF-β1 for 21 days. **(e)** The vector- and G3-transfected MC3T3-E1 cells were maintained in 10% FBS/AMEM medium with or without 1 ng/ml TGF-β1 for 3 days. All cells were lysed and subjected to immunoblotting with antibodies to pEGFR, pAKT, pSAPK/JNK, GSK-3β (S9P) and β-actin.

In the present study, we stably transfected MC3T3-E1 cells with a G3 construct and observed that G3 expressing MC3T3-E1 cells inhibited cell growth in the presence of TGF-β1 (1 ng/ml), compared with the vector control cells (Figure 3a, Figure 3b, Additional file 3: Figure S3a, and Additional file 3: Figure S3b). Versican G3-expressing MC3T3-E1 cells also showed lower ALP activity compared with the vector control cells. Thus versican appeared to inhibit MC3T3-E1 cell differentiation in the presence of TGF-β1 (Figure 3c and Figure 3d). Immunoblotting showed that G3-expressing MC3T3-E1 cells upregulated pEGFR and pAKT. When cultured in TGF-β1(1 ng/ml), G3-expressing MC3T3-E1 cells also showed increased levels of pSAPK/JNK, pAKT and decreased levels of GSK-3β (S9P) (Figure 3e).

Versican G3 domain promotes cell proliferation in breast cancer and many other carcinoma cells *in vitro* and *in vivo*[22]. G3-expressing breast cancer cells showed drug resistance to Doxorubicin and Epirubicin, but expressed enhanced apoptosis when cultured in C2-ceramide and Docetaxel [23,24]. Versican and its G3 domain inhibited mesenchymal chondrogensis through mechanisms involving its EGF-like motifs [48]. The present research shows that G3 inhibits osteoblast cell growth and differentiation in TGF-β1 conditioned medium and promotes cell apoptosis induced by TGF-α. Versican is highly expressed in advanced breast cancer patients, as is TGF-β and TGF-α, indicating that the interaction of these molecules may facilitate tumor cell haptotactic migration towards bony tissues.

**Figure 4:**
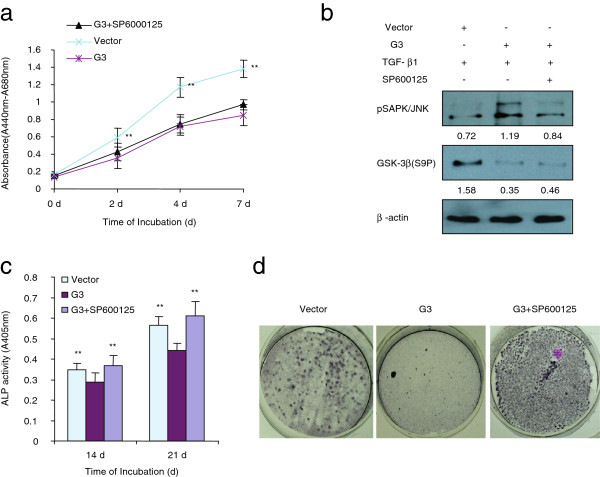
** Selective SAPK/JNK inhibitor SP600125 blocks versican G3 enhanced MC3T3-E1 cell apoptosis but doesn’t have effect on G3 inhibited cell growth.(a)** The G3- and vector-transfected MC3T3-E1 cells (1 × 10^3^) were inoculated in 96-well culture dishes and cultured in AMDM medium containing 1 ng/ml TGF-β1 with or without 100 nM SP60025 for 7 days. Proliferation assays performed with WST-1 assay. All groups compared with vector control cells, *n* = 8, * p<0.05, ** p<0.01, analyzed with *t*-test. **(b)** The G3- and vector-transfected MC3T3-E1 cells (1 × 10^5^) were cultured in AMDM medium containing 1 ng/ml TGF-β1 with or without 100 nM SP60025 for 24 hours. All samples were lysed and subjected to immunoblotting with antibodies to pSAPK/JNK, GSK-3β (S9P) and β-actin. **(c)** The vector- and G3-transfected MC3T3-E1 cells were maintained in 10% FBS/AMEM medium with or without 100 nM SP60025 for 21 days. The medium was changed every 3 days. Cells were lysed and processed to ALP ELISA Assay. All groups compared with vector control cells, *n* = 6, * p<0.05, ** p<0.01, analyzed with *t*-test. **(d)** Typical pictures showed ALP staining of the samples.

When cultured in TGF-β, the G3-expressing MC3T3-E1 cells showed inhibited cell growth and differentiation, and expressed increased expression levels of pSAPK/JNK and decreased levels of GSK-3β (S9P). When cultured in TNF-α, the G3-expressing MC3T3-E1 cells showed enhanced cell apoptosis induced by TNF-α, and expressed increased expression levels of pSAPK/JNK without appreciable changes to GSK-3β (S9P) expression. To observe whether enhanced pSAPK/JNK expression resulted in the alteration in proliferation and differentiation in G3-expressing MC3T3-E1 cells, we cultured the G3-expressing MC3T3-E1 cells with one of the selective SAPK/JNK inhibitors SP600125. We found that it did not block G3 inhibition of cell growth in the presence of TGF-β (Figure 4a). However, selective SAPK/JNK inhibitor SP600125 could prevent G3 inhibitory effects on MC3T3-E1 cell differentiation (Figure 4c and Figure 4d). Immunoblotting confirmed that selective SAPK/JNK inhibitor SP600125 prevented G3 enhanced expression levels of pSAPK/JNK and had no effect on decreased GSK-3β (S9P) expression, when the cells were cultured in TGF-β medium (Figure 4b). These results indicate that versican G3 domain can enhance the inhibition of MC3T3-E1 cell differentiation in the presence of TGF-β through enhanced expression of EGFR/JNK signaling. Selective SAPK/JNK inhibitor SP600125 blocked G3 enhanced expression of EGFR/JNK signaling in MC3T3-E1 cells, and as a result, prevented its inhibition on cell differentiation. On the other hand, selective SAPK/JNK inhibitor SP600125 did not prevent expression of versican G3 enhanced cell growth inhibition induced by TGF-β, indicating that versican G3 enhanced inhibition of MC3T3-E1 cell growth induced by TGF-β was not related with its enhanced EGFR/JNK activity, and may be related with other factors, such as down-regulation of GSK-3β (S9P) expression.

Tumor necrosis factor alpha (TNF-α) is a pleiotropic cytokine that plays an important role in immunity and inflammation as well as in the control of cell proliferation, differentiation, and apoptosis [31]. TNF-α is produced mainly by macrophages and enhances tumor regression mediated by cytotoxic T cells. TNF-α has been implicated to play a role in advanced breast cancer and some other metastatic tumors. It induces tumor necrosis by initiating apoptotic cell or death affecting tumor vascularization [49]. Paradoxically however, it can also promote tumor cell proliferation and progression [50,51].

**Figure 5:**
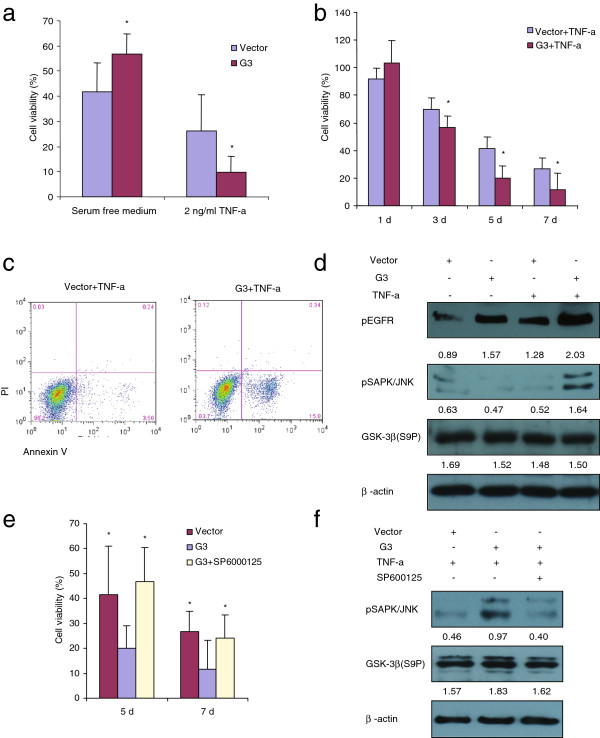
** Expression of versican G3 domain regulated MC3T3-E1 cells apoptosis via EGFR/JNK pathway.(a)** G3- and vector-transfected MC3T3-E1 cells (2 × 10^5^) were inoculated in 6 well culture dishes with 10% FBS/AMEM medium. After cultured for 12 hours, all samples were treated serum free AMEM medium with or without 2 ng/ml TNF-α for 4 days. The survival cells were counted under light microscope and compared with the seeded cells to present the viability (%). All groups compared with vector control cells, *n* = 6, * p<0.05, ** p<0.01, analyzed with *t*-test. **(b)** G3- and vector-transfected MC3T3-E1 cells (1 x 10^3^) were inoculated in 96-well culture dishes and cultured in 10%FBS/AMEM medium for 12 h. All samples were treated serum free AMEM medium with or without 2 ng/ml TNF-α for 7 days. All groups compared with vector control cells, *n* = 9, * p<0.05, ** p<0.01, analyzed with *t*-test. The cell viability was assayed by WST-1 assays. **(c)** G3- and vector-transfected MC3T3-E1 cells were treated with serum free AMEM medium with 2 ng/ml TNF-α for 24 hours and processed to Annexin V assays. **(d)** G3- and vector-transfected MC3T3-E1 cells were treated with serum free AMEM medium with 2 ng/ml TNF-α for 24 hours. All samples were lysed and subjected to immunoblotting with antibodies to pEGFR, pSAPK/JNK, GSK-3β (S9P) and β-actin. **(e)** G3- and vector-transfected MC3T3-E1 cells were treated with serum free AMEM medium with 2 ng/ml TNF-α, with or without 100 nM SP60025 for 4 days. Cell viability was assessed by WST-1 assays. The cell viability was presented by absorbance value of treated samples compared with absorbance value of same groups 12 hours after loading (%). The survived cells were counted under light microscope and compared with the seeded cells to present the viability (%). **(f)** G3- and vector-transfected MC3T3-E1 cells were treated with serum free AMEM medium with 2 ng/ml TNF-a, with or without 100 nM SP60025 for 24 hours. All samples were lysed and subjected to immunoblotting with antibodies to pSAPK/JNK, GSK-3β (S9P) and β-actin.

In this study, we found that versican G3-expressing MC3T3-E1 cells showed enhanced cell survival in serum free AMEM medium, while lower cell viability was observed in serum free AMEM medium with TNF-α (2 ng/ml) compared to vector control cells (Figure 5a, Figure 5b and Additional file 4: Figure S4). Annexin V-FITC apoptosis detection assays confirmed that versican G3-expressing MC3T3 E cells showed enhanced cell apoptosis in serum free AMEM medium with TNF-α (2 ng/ml) when compared to vector cells (Figure 5c). Immunoblotting showed that G3-expressing MC3T3-E1 cells expressed enhanced pEGFR in serum free AMEM medium with or without TNF-α (2 ng/ml). When cultured in TNF-α (2 ng/ml), G3-expressing MC3T3-E1 cells also showed increased expression of pSAPK/JNK, although GSK-3β (S9P) expression did not appear influenced (Figure 5d). Selective SAPK/JNK inhibitor SP600125 could also prevent versican G3 enhanced MC3T3-E1 cell apoptosis induced by TNF-α (Figure 5e). SP6000125 blocked G3 enhanced expression levels of pSAPK/JNK and had no effect on GSK-3β (S9P) expression, when the cells were cultured in TNF-α medium (Figure 5f). These results indicated that versican G3 domain enhanced MC3T3-E1 cell apoptosis induced by TNF-α through enhanced expression of EGFR/JNK signaling. Selective SAPK/JNK inhibitor SP6000125 blocked G3 enhanced expression of EGFR/JNK signaling observed in MC3T3-E1 cells and thereby prevented its enhanced effect on pre-osteoblast cell apoptosis.

### Versican G3 domain modulated MC3T3-E1 cell differentiation, growth and apoptosis through epidermal growth factor-like motifs

**Figure 6:**
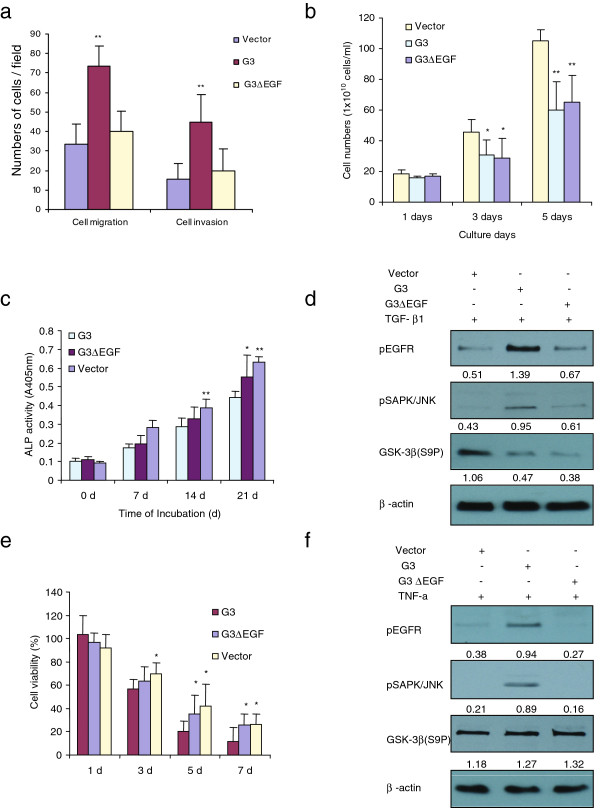
** Versican G3 domain modulated MC3T3-E1 cell differentiation, growth and apoptosis via its epidermal growth factor-like motifs.(a)** After culture the MC3T3-E1 cells in the bottom well of Transwell migration chambers for 12 h, vector, G3- and G3ΔEGF- transfected 4T07 cells (1 × 10^5^) were loaded in the insert pre-set with matrigel gel with 100 μl serum free DMEM medium and then incubated at 37°C for 48 hours. The invasive cells were stained blue and were counted in 6 fields of views/membrane using a light microscope. **, p<0.01. Error bars indicate SD (n = 6). **(b)** Vector-, G3- and G3ΔEGF- transfected MC3T3 cells (2 × 10^4^) were inoculated in 6-well culture dishes containing 10% FBS/AMEM and cultured for 12 h. After cell attachment, the cells were cultured with 1 ng/ml TGF-β1 for 5 d. Cells were counted by light microscope in 1, 3, 5 days. *n* = 6, * p<0.05, ** p<0.01, analyzed with *t*-test. **(c)** The G3- and G3ΔEGF-, and vector- transfected MC3T3-E1 cells were seeded at 8 × 10^4^ cells/well in a 6 well plate. Cells were maintained in 10% FBS/AMEM medium for 21 days. The medium was changed every 3 days. Cells were lysed and processed to ALP ELISA Assay. All groups compared with vector control cells, *n* = 6, * p<0.05, ** p<0.01, analyzed with *t*-test. **(d)** The G3- and G3ΔEGF- transfected MC3T3-E1 cells were maintained in 10% FBS/AMEM medium with 1 ng/ml TGF-β1 for 3 days. All cells were lysed and subjected to immunoblotting with antibodies to pEGFR, pSAPK/JNK, GSK-3β (S9P) and β-actin. **(e)** The G3- and G3ΔEGF-, and vector- transfected MC3T3-E1 cells (1 × 10^3^) were inoculated in 96-well culture dishes and cultured in 10%FBS/AMEM medium for 12 h. All samples were treated serum free AMEM medium with or without 2 ng/ml TNF-α for 7 days. The survival cells were counted under light microscope and compared with the seeded cells to present the viability (%). All groups compared with vector control cells, *n* = 9, * p<0.05, ** p<0.01, analyzed with *t*-test. The cell viability was assayed by WST-1 assays. **(f)** The G3- and G3ΔEGF- transfected MC3T3-E1 cells were treated with serum free AMEM medium with 2 ng/ml TNF-α for 24 hours. All samples were lysed and subjected to immunoblotting with antibodies to pEGFR, pSAPK/JNK, GSK-3β (S9P) and β-actin.

There appears to be important functions of the EGF-like motifs of versican G3 domain [52]. In transiently transfected breast cell lines 66c14 and 4T07 with G3 fragment lacking the EGF-like motifs (G3ΔEGF), the G3ΔEGF expressing cells did not show enhanced cell growth and migration when compared to G3 transfected cells. We also stably transfected these constructs into 4T07 cells, and found that G3 expressing breast cancer cells showed enhanced cell migration and invasion to MC3T3-E1 cells. But the G3ΔEGF expressing cells did not show enhanced cell migration and invasion to MC3T3-E1 cells (Figure 6f).

In our experiments, we also stably transfected MC3T3-E1 cells with a G3 construct, G3ΔEGF, and vector. We found that G3ΔEGF-expressing MC3T3-E1 cells did not show enhanced cell growth inhibition induced by TGF-β1 when compared to the G3 transfected cell group (Figure 6b, 6c). The EGF like motifs of G3 domain did not appear to be one of the main participants in the TGF-β-induced growth inhibition of MC3T3E1 cells. However the EGF repeats were demonstrated to play an important role in TGF-β-induced inhibition of cell differentiation. G3ΔEGF-expressing MC3T3-E1 cells did show enhanced cell differentiation in TGF-β1 medium when compared with the G3 transfected cell group in 21 days (Figure 6c). Immunoblotting experiments showed that G3ΔEGF expressing cells did not show enhanced pEGFR and pSAPK/JNK as compared to G3 transfected cells but did express decreased levels of GSK-3β (S9P), as G3 transfected cells did in TGF-β CM (Figure 6d). G3ΔEGF-expressing MC3T3-E1 cells did not show enhanced cell growth apoptosis induced by TNF-α when compared to the G3 transfected cell group (Figure 6e). Immunoblotting showed that G3ΔEGF expressing cells did not show enhanced pEGFR and pSAPK/JNK expression as G3 transfected cells did in serum free AMEM medium containing TNF-α (Figure 6f).

In summary, dependency on EGF-like motifs in versican G3 was observed in G3’s ability to enhance inhibition of MC3T3-E1 cell differentiation induced by TGF-β and cell apoptosis induced by TNF-α. Without the structure of its EGF-like repeats, G3 domain lost its function in activating the EGFR/JNK signaling pathway, and thus did not confer its previously observed ability to inhibit MC3T3-E1 cell differentiation and promote MC3T3-E1 cell apoptosis.

### The potential mechanisms by which versican enhances breast cancer cell metastasis to bone

Specific aspects of breast cancer cells, tumor stroma, and the bone microenvironment contribute to the development of bone metastasis. Breast cancer preferentially spreads to bone [53]. Tumor cells can produce or stimulate tumor stromal cells to secrete a variety of cytokines, ECM components (such as versican), and other bioactive factors that act on cells in the tumor, stroma and bone. Given an appropriate environment, tumor cells become more invasive, stromal tissues support tumor outgrowth, and metastasis occurs. The bone microenvironment favors tumor cell colonization for cancers such as breast, prostate, lung, renal, and colon [54]. Breast cancer metastasis is historically bone destructive and osteolytic in nature, although recent systemic advances in therapy including bisphosphonates that potently inhibit osteoclastic activity has resulted in more mixed osteolytic/osteoblastic disease. Thus, the specific molecular interactions between the breast cancer cells, stromal tissues and the bone microenvironment drive the development of bone metastasis. A mechanistic understanding of the molecular factors associated with poor prognosis is important in developing new therapies and molecular targets [55].

Local and systemic immune modulators influence the tumor phenotype [42,43]. Several cytokines and growth factors participate in tumor-stroma connectivity, in particular transforming growth factor-β (TGF-β) and tumor necrosis factor-α (TNF-α). These factors are initially stimulated by the immune system in response to tumor cells, playing an important role in both immunity and inflammation. These factors have also been shown to regulate tumor/stromal cell proliferation, differentiation, and apoptosis [31]. During the early stages of tumorigenesis, TGF-β inhibits tumor growth, and TNF-α induces tumor necrosis by initiating apoptotic cell or death affecting tumor vascularization. Paradoxically however, they can also promote tumor cell proliferation, progression and metastasis in advanced breast cancer [50]. Thus, both TNF-α and TGF-β display a dual role in breast cancer tumorigenesis – both as tumor promoters and as tumor suppressors [31].

**Figure 7:**
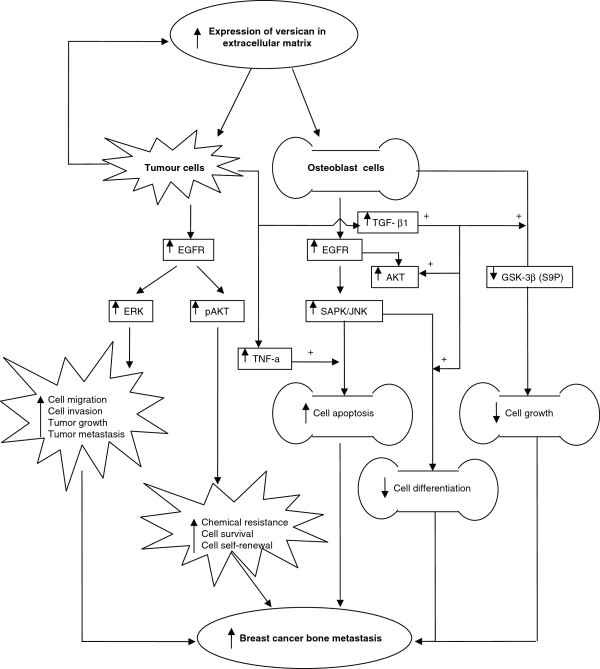
** The potential mechanisms that versican enhances breast cancer cell metastasize to bone.** The interaction between breast cancer cells and tumor stroma induce tumor cells and stromal cells expressing enhanced cytokins TGF-β 1, TNF-α, and extracellular matrix – versican. Enhanced expression of versican promotes tumor cells expressing enhanced levels of pEGFR, pERK, and pAKT. Expression of ERK enhances tumor cell migration, invasion, growth, and metastasis. While expression of pAKT enhances tumor cell chemical resistance, cell survival, cell self-renewal. On the other hand, Over-expression of versican and TGF-β promote osteoblast cells expressing enhanced EGFR/JNK, which inhibits osteoblast cell differentiation. Elevated expression levels of versican and TNF-α in bone stroma activate pEGFR /pJNK signaling in osteoblast cells, which induces ostoblast cells apoptosis. Versican and TGF-β related inhibited of ostoblast growth may partially releted to downregulated expression of GSK-3β (S9P). In summary, versican enhances breast cancer bone metastasis not only by enhancing tumor cell mobility, invasion, and survival in bone tissues, but also via inhibiting osteoblast cell growth, differentiation, which supply favorable microenvironments for tumor metastasis.

Breast cancer stromal cells express enhanced TGF-β-1, TNF-α, and extracellular matrix molecules such as versican. Enhanced versican expression promotes enhanced levels of pEGFR, pERK, and pAKT. Expression of pERK enhances tumor cell migration, invasion, growth, and metastasis. We have previously shown that expression of pAKT enhances tumor cell resistance to certain chemotherapeutics and influences cellular survival and self-renewal. In this study, the over-expression of versican and TGF-β promoted pre-osteoblast cell expression, enhancing EGFR/JNK signaling. This subsequently inhibited osteoblast cell differentiation. Enhanced expression of versican and TNF-α in bone stroma activated pEGFR/pJNK signaling in osteoblast cells, which induced osteoblastic cell apoptosis. The differential influence of versican G3 on breast cancer cells and osteoblasts may depend on activated expression of EGFR signaling and its downstream pathways (Figure 7). The EGFR downstream pathway protein GSK-3β (S9P) is upregulated in versican G3 expressing breast cancer cells, and downregulated in G3 expressing osteoblasts.

## Conclusions

In summary, the results of this *in-vitro* study demonstrate that versican enhances tumor cell mobility, invasion, and survival in bone tissues. It also acts as an inhibitor of bone stromal and pre-osteoblast MC3T3-E1 cell growth. This may explain in part, why the bone acts as a favorable microenvironment for breast cancer cell metastasis. Versican and its related G3 domain with its EGF-like motifs influence downstream EGFR and AKT signaling, influencing bone stromal and pre-osteoblast cells. It also appears to modulate TGF-β-1 and TNF-α bone related activity.

## Competing interests

The authors declare that they have no competing interests.

## Authors’ contributions

The authors’ contributions to this research work are reflected in the order shown. WWD contributed to the majority of the experimental work and writing the manuscript. AJY and BBY directed the research, designed and coordinated the project, analyzed the data, and wrote the manuscript. WS, YZ and AS conceived the study and participated in its design. LF and WY were involved in flow cytometry assays and data analysis. All authors read and approved the final manuscript.

## Pre-publication history

The pre-publication history for this paper can be accessed here:

http://www.biomedcentral.com/1471-2407/12/341/prepub

## Supplementary Material

Additional file 1 ** Figure S1.** (a) MC3T3 cells (2 × 10^4^) were inoculated in 6-well culture dishes containing 10% FBS/AMEM and cultured for 12 h. After cell attachment, we changed the medium to MC3T3-E1, 67NR, 66c14, 4T07, and 4T1 CM for 2 d, and kept culture for 5 d. Typical pictures showed that the medium pre-inculbated with 4T1 cells inhibits MC3T3-E1 cell growth. (b) The MC3T3-E1 cells were seeded at 8 × 10^4^ cells/well in 6 well plates. Cells were maintained in MC3T3-E1, 67NR, 66c14, 4T07, and 4T1 CM for 21 days. The medium was changed every 3 d. After 21 d, all samples were processed to Alkaline Phosphatase ELISA staining. (c) Modified chemotactic Boyden chamber migration assays indicated that 4T1 cell line showed highest motility compared with other mouse breast cancer cell lines after migrated for 4 h. Compared with 67NR cell line, *n* = 4, * p<0.05, ** p<0.01, analyzed with *t*-test. (d) Immunoblotting showed that 4T1 cells expressed highest level of versican V1 isoform (250 KD). Click here for file

Additional file 2 ** Figure S2.** (a) Typical pictures showed that migration cells of 67NR, 66c14, 4T07, 4T1 cell lines after 4 h cell migration. (b) Typical pictures showed that invasive cells of 67NR, 66c14, 4T07, 4T1 cell lines 48 h after cell invasion assay. Click here for file

Additional file 3 ** Figure S3.** (a) Immunoblotting showed that G3 transfected MC3T3-E1 cells expressed highest level of G3 protein. (b)Vector- and G3- transfected MC3T3 cells (2 × 10^4^) were inoculated in 6-well culture dishes containing 10% FBS/AMEM and cultured for 12 h. After cell attachment, the cells were cultured with or without 1ng/ml TGF-β1 for 5 d. Typical pictures showed the cells after 4 days culture. Click here for file

Additional file 4 ** Figure S4.** Typical pictures showed that G3- and vector-transfected MC3T3-E1 cells treated with serum free AMEM medium with or without 2 ng/ml TNF-a for 5 days. Click here for file
