# Supplementary figures and images for: The role of versican G3 domain in regulating breast cancer cell motility including effects on osteoblast cell growth and differentiation in vitro – evaluation towards understanding breast cancer cell bone metastasis
Source: BMC Cancer. 2012 Aug 3;12:341. doi: 10.1186/1471-2407-12-341 (PMC3489894; doi:10.1186/1471-2407-12-341)

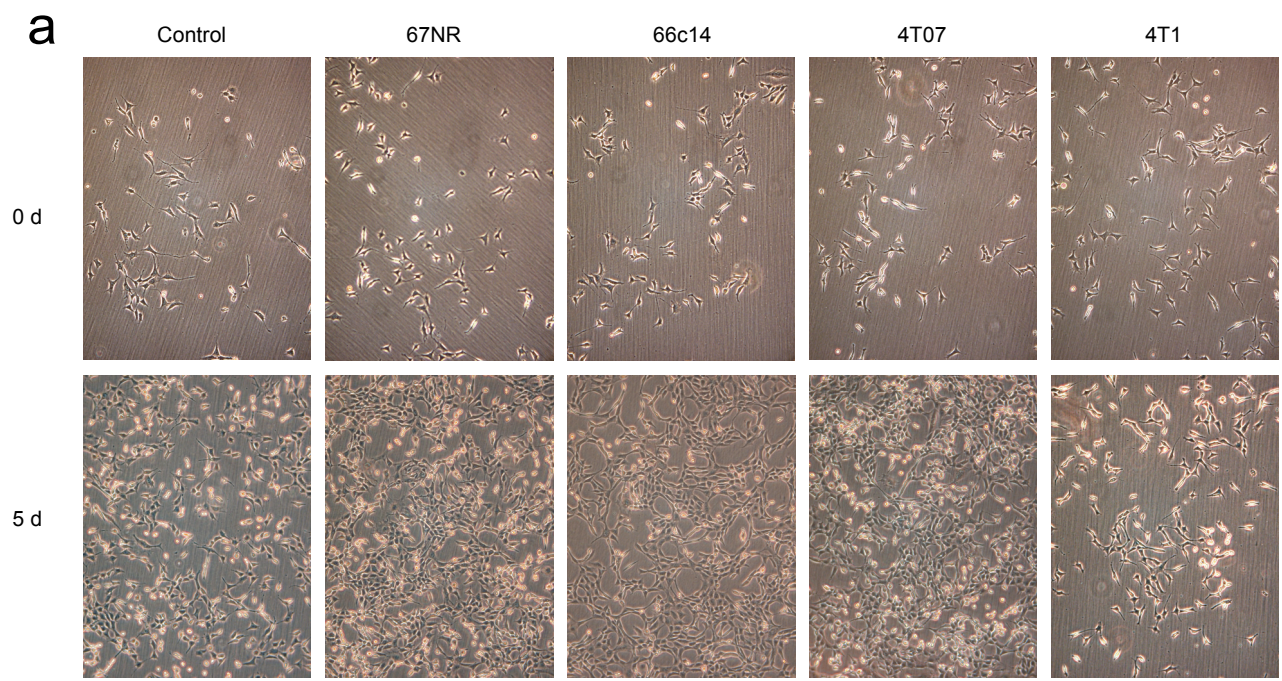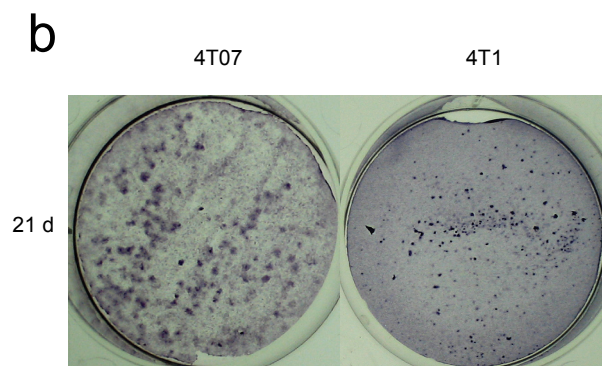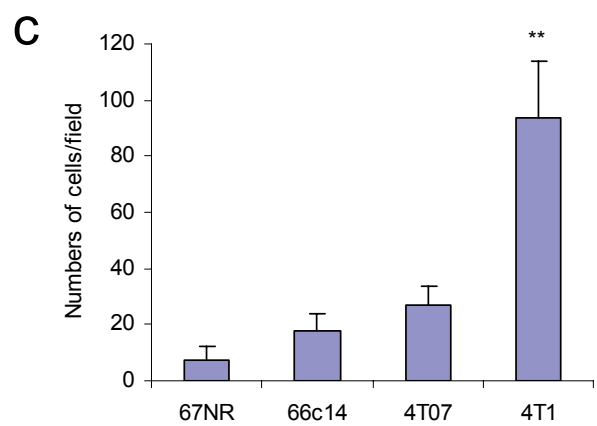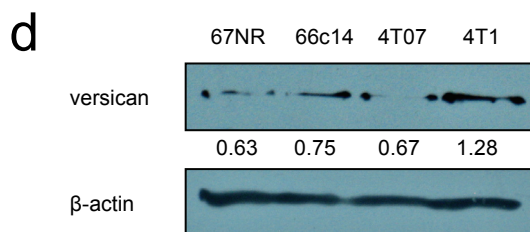

Figure S1

Supplement: Additional file 1 — Figure S1. (a) MC3T3 cells (2 × 10^4^) were inoculated in 6-well culture dishes containing 10% FBS/AMEM and cultured for 12 h. After cell attachment, we changed the medium to MC3T3-E1, 67NR, 66c14, 4T07, and 4T1 CM for 2 d, and kept culture for 5 d. Typical pictures showed that the medium pre-inculbated with 4T1 cells inhibits MC3T3-E1 cell growth. (b) The MC3T3-E1 cells were seeded at 8 × 10^4^ cells/well in 6 well plates. Cells were maintained in MC3T3-E1, 67NR, 66c14, 4T07, and 4T1 CM for 21 days. The medium was changed every 3 d. After 21 d, all samples were processed to Alkaline Phosphatase ELISA staining. (c) Modified chemotactic Boyden chamber migration assays indicated that 4T1 cell line showed highest motility compared with other mouse breast cancer cell lines after migrated for 4 h. Compared with 67NR cell line, n = 4, * p<0.05, ** p<0.01, analyzed with t-test. (d) Immunoblotting showed that 4T1 cells expressed highest level of versican V1 isoform (250 KD). [file 1471-2407-12-341-S1.pdf]

a

67NR

66c14

4T07

4T1

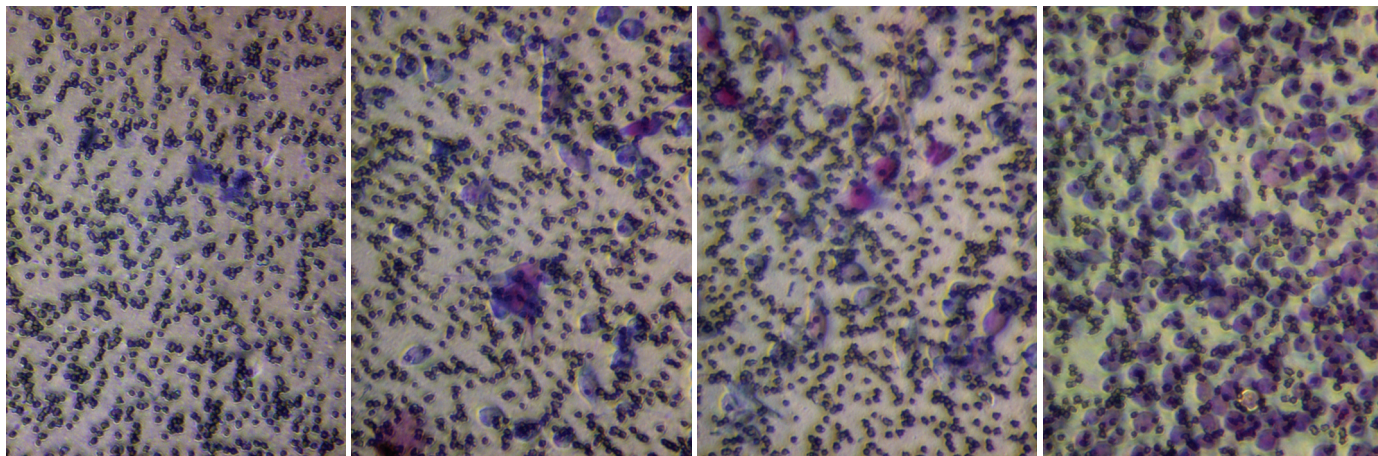

b

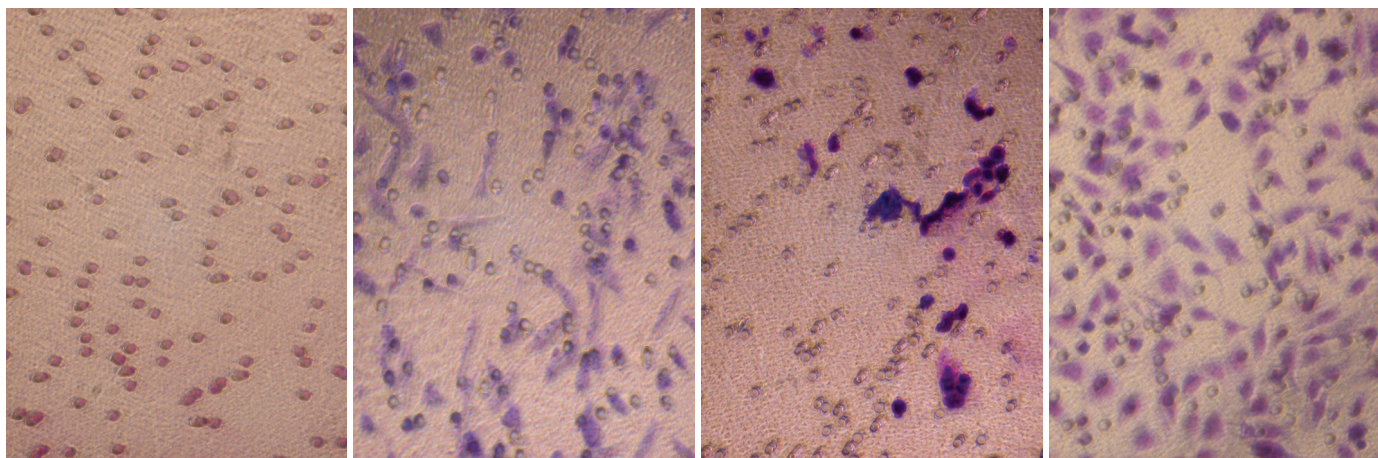

Figure S2

Supplement: Additional file 2 — Figure S2. (a) Typical pictures showed that migration cells of 67NR, 66c14, 4T07, 4T1 cell lines after 4 h cell migration. (b) Typical pictures showed that invasive cells of 67NR, 66c14, 4T07, 4T1 cell lines 48 h after cell invasion assay. [file 1471-2407-12-341-S2.pdf]

**a**

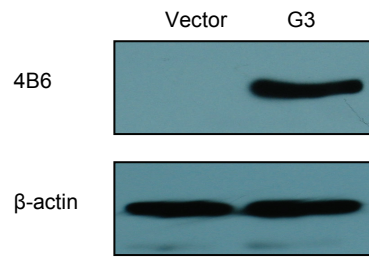

**b**

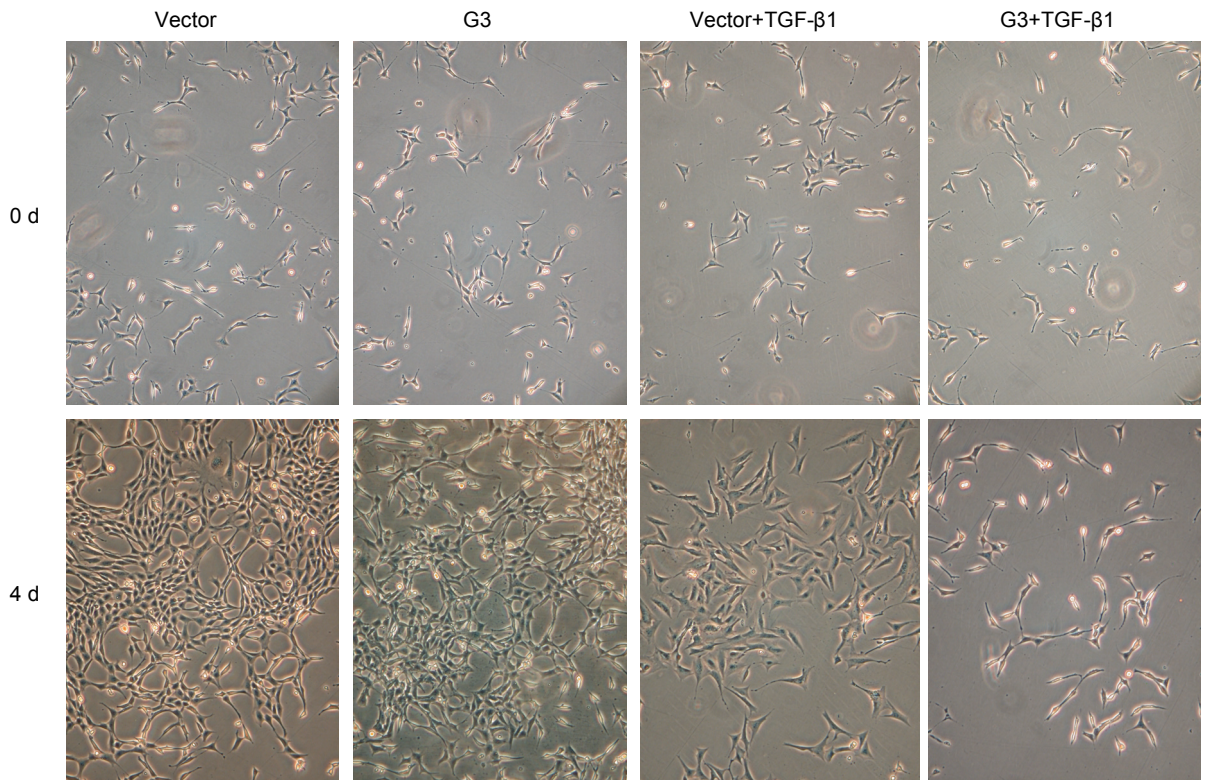

Figure S3

Supplement: Additional file 3 — Figure S3. (a) Immunoblotting showed that G3 transfected MC3T3-E1 cells expressed highest level of G3 protein. (b)Vector- and G3- transfected MC3T3 cells (2 × 10^4^) were inoculated in 6-well culture dishes containing 10% FBS/AMEM and cultured for 12 h. After cell attachment, the cells were cultured with or without 1ng/ml TGF-β1 for 5 d. Typical pictures showed the cells after 4 days culture. [file 1471-2407-12-341-S3.pdf]

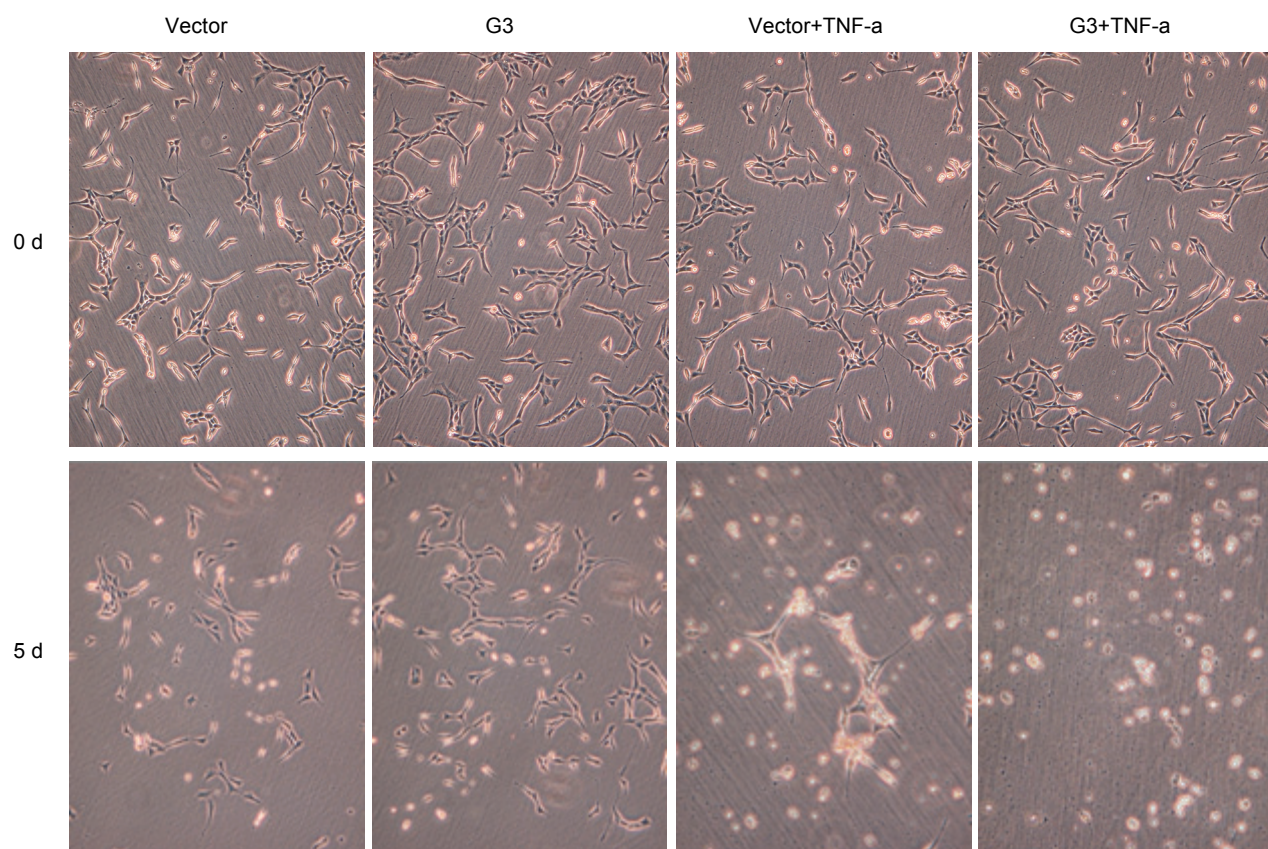

Figure S4

Supplement: Additional file 4 — Figure S4. Typical pictures showed that G3- and vector-transfected MC3T3-E1 cells treated with serum free AMEM medium with or without 2 ng/ml TNF-a for 5 days. [file 1471-2407-12-341-S4.pdf]
